# Book Notices

**Published:** 1879-12-20

**Authors:** 


### *BOOK NOTICES.*

DISEASES OF WOMEN, by LAWSON TAIT, F. R. C. S., Surgeon to the Birmingham Hospital for Women, and Consulting Surgeon to the West Broomwich Hospital, Fellow of the Obstetric Society, etc., etc. Second edition, thoroughly revised and enlarged, specially prepared for Wood's library in New York: Wood & Co, 27 Great Jones Street. 1879.

This is a practical work of 186 octavo pages, in which the author gives many valuable suggestions, drawn from his own experience.

MEMORIAL ORATION IN HONOR OF EPHRIAM McDOWELL, the father of Ovariotomy, by SAMUEL D. GROSS, M. D., L. L. D., D. C. L. Oxon. Delivered at Danville, Ky., at the dedication of the monument erected to the memory of Dr. Ephriam McDowell by the Kentucky State Medical Society, May 14, 1879. Published by the Society, Louisville, Ky.; printed by John P. Morton & Co. 1879.

TRANSACTIONS OF THE MEDICAL SOCIETY OF THE STATE OF PENNSYLVANIA, at the Thirteenth Annual Session, held at Chester, May, 1879. Vol XII. Part II. Published by the Society. Philadelphia.

The Pennsylvania Society is perhaps the largest State organization in the Union, numbering over seven hundred permanent members, many of whom are at the very head and front of the profession on this continent.

The present volume numbers nearly a thousand pages, and contains a number of very interesting and valuable papers. Among them are the addresses of James L. Stewart, M. D., the President; Chas. T. Hunter, M. D.; the papers of Wm. Goodell, M. D.; Andrew Fleming, M. D.; John H. Packard. M. D.; P. J. Lewis, M. D.; P. D. Keyser, M. D.; Wm. Pepper, M. D.; Richard A. Cleeman, M. D.; C. S. Turnbull, M. D.; Oscar H. Allis, M. D.; Ellwood Harvey, M. D.; John V. Shoemaker, A. M., M. D.; Benjamin Lee, M. D.

The reports of numerous county societies are also contained in the volume, in which are much instructive and valuable matter.

INFANT FEEDING AND ITS INFLUENCE ON LIFE, OR THE CAUSES AND PREVENTION OF INFANT MORTALITY, by C. H. F. Routh, M. D , M. R. C. P. L. Fellow of University College, London, of the Medical, Medico-Chirurgical and Obstetrical Societies; Corresponding member of the Royal Academy of Madrid and Pesth, and the Gynæcological Society of Boston; Senior Physician to the Samaritan Hospital for Women aud Children, etc. Third edition. New York: Wm. Wood & Co, 37 Great Jones street. 1879.

A valuable work of two hundred and seventy pages upon a much neglected and very important department of Medical science.

---

### *ANALYTICAL CHEMISTRY.*

*Prof. E. J. Hallock's Card.*—We invite attention to the card of E. J. Hallock, Professor of Chemistry in the Southern Medical College. He is a gentleman of eminent ability, and has opened an office in the Medical College building, and is prepared to make tests and analyses of every kind.

